# Overexpression of G6PD is associated with high risks of recurrent metastasis and poor progression-free survival in primary breast carcinoma

**DOI:** 10.1186/s12957-015-0733-0

**Published:** 2015-11-25

**Authors:** Haihong Pu, Qingyuan Zhang, Chunbo Zhao, Lei Shi, Yan Wang, Jingxuan Wang, Minghui Zhang

**Affiliations:** Department of Medical Oncology, The Third Affiliated Hospital of Harbin Medical University, Haping Road 150 of Nangang District, Harbin, Heilongjiang Province 150086 China; Department of radiation oncology, The Fourth Affiliated Hospital of Harbin Medical University, Harbin, Heilongjiang Province 150001 China

**Keywords:** G6PD, Primary breast carcinoma, Prognosis, Immunohistochemistry

## Abstract

**Background:**

The present study aimed to investigate the expression of CYP27A1, CYP7B1, insulin-like growth factor-1 (IGF-1), glucose-6-phosphate-dehydrogenase (G6PD), glutathione S-transferase P1 (GSTP1), and pyruvate kinase isoform M2 (PKM2) in breast carcinoma tissue and evaluate their prognostic value for progression-free survival (PFS) and overall survival (OS).

**Methods:**

A total of 20 patients treated with surgery for primary breast carcinoma were enrolled: 10 cases diagnosed with recurrent metastasis (A), along with their corresponding metastases specimen (A_M_) and 10 cases with no evidence of recurrence or metastasis (B). Baseline characteristics of patients including age, lymph node metastasis, molecular subtypes, tumor staging and size, and pathological classification were all collected. Immunohistochemistry was performed to detect the protein expression in tumor specimens.

**Results:**

Elevated G6PD protein levels were noted in group A compared with group A_M_ and B (both *P* < 0.05), and PKM2 expression was also higher in group A when compared to group A_M_ (*P* = 0.019), but similar with group B (*P* > 0.05). No association between clinicopathological parameters and the two proteins expression was observed. The G6PD protein expression was strongly associated with PFS of breast carcinoma patients (*P* = 0.021) but not for OS. According to the Kaplan-Meier analysis, mean PFS time of patients with G6PD-negative and G6PD-positive expression tumor were 71.36 ± 6.53 and 32.25 ± 5.67 months, respectively (*P* = 0.002).

**Conclusions:**

The G6PD protein could be served as a potential prognostic biomarker for primary breast carcinoma, and overexpression of G6PD protein predicted a high risk of recurrent metastasis and poor PFS during follow-up.

## Background

Breast carcinoma is the most frequent malignant tumor in female and accounts for more than 1,000,000 new cases annually [1]. It has been the second leading cause of carcinoma-related death for female overall [2], with an increasing mortality rate worldwide during the past 60 years [3]. Recently, the knowledge of cellular and molecular characteristics in breast carcinoma has facilitated a shift toward the development of carcinoma diagnosis and treatment [4, 5]. However, despite the new advances in the treatment of breast carcinoma, such as the increasing application of surgery combined with neoadjuvant chemotherapy or hormone therapy [6], its prognosis is poor as the risk of recurrence or metastasis is ever present [7]. Hence, it is imperative to delineate the molecular mechanisms underlying the recurrence or metastasis of breast carcinoma.

Previously, España et al. has reported an association between the metastatic activity of the cancer cells and the regulation of glycometabolism and amino acid metabolism during his study which focused on the interaction of proteins in this carcinoma cells transfected with Bcl-x (L) [8]. More recently, Shidfar al. also demonstrated that lipid metabolism genes in tumor and contralateral unaffected breast were conversely relative to the status of tumor estrogen receptor [9].

However, few researches focusing on the glycometabolism- and lipid metabolism-related protein expression in breast carcinoma were available. Hence, this study aimed to investigate the expression of glycometabolism- and lipid metabolism-related proteins in breast carcinoma, including CYP27A1, CYP7B1, insulin-like growth factor-1 (IGF-1), glucose-6-phosphate-dehydrogenase (G6PD), glutathione S-transferase P1 (GSTP1), and pyruvate kinase isoform M2 (PKM2), and to evaluate their significance in the prognosis of this disease.

## Methods

### Patients

A total of 20 patients treated with total mastectomy for primary breast carcinoma in our hospital between January 2005 and January 2014 were enrolled. The inclusion criteria were as follows: (i) breast carcinoma pathologically diagnosed with recurrent metastasis during the follow-up (A, *n* = 10), along with their corresponding metastasis tissue sample (A_M_, *n* = 10) and (ii) breast carcinoma with no pathological evidence of local recurrence or metastasis during the follow-up (B, *n* = 10). All the patients were postmenopausal female. They all underwent chemotherapy or radiotherapy as well as endocrine therapy with arimedex after surgery. The metastasis sites included the right mammary gland, infraclavicula, and chest wall or accompanied with the lung, liver, neck, or bone. The baseline characteristics of patients including age, lymph node metastasis, molecular subtypes, tumor staging and size, as well as pathological classification were all collected. Primary breast carcinoma specimens of groups A and B were sampled before chemotherapy/radiotherapy, and the metastasis specimens were collected from patients who had stopped chemotherapy/radiotherapy for at least 1 year before their recurrence during the follow-up.

### Immunohistochemistry

Immunohistochemical staining was performed to detect the expression of CYP27A1, CYP7B1, IGF-1, G6PD, GFPT1, and PKM2 in tumor tissues. Formalin-fixed paraffin sections (4-μm thick) of tumor specimen were deparaffinized at 58 °C for 24 h followed by xylene I, II, and III for 10 min, respectively, and rehydrated in a descending series of alcohol (95, 85, and 75 % for 2 min, respectively) and then washed three times with phosphate-buffered saline (PBS) for 3 min. Then, they were heated twice for antigen retrieval in 10 mM citrate buffer solution (pH 6.0) in a microwave oven (98 °C) for 10 min. After returning to room temperature, the sections were incubated with 1 % H_2_O_2_ for 20 min or 3 % H_2_O_2_ for 5–10 min to eliminate the endogenous peroxidase activity before rinsing three times in PBS for 3 min. Thereafter, they were blocked with biotinylated goat anti-rabbit immunoglobulins (Abcam) overnight at 4 °C following rinsing three times in PBS for 3 min. After incubation with Envision reagent for 30 min at 37 °C and rinsing three times in PBS for 3 min, staining was revealed using 0.04 % 3,3′-diaminobenzidine (DAB) substrate and 0.03 % H_2_O_2_ following tap water washing for 3 min and then counterstained with hematoxylin for 30 s, washing, hydrochloric acid-ethanol for 2 s, and washing. Staining with PBS in place of primary antibody was used as negative control. The positive staining in tumor cells exhibited buffy or brown color with blue as background.

The percentage of stained tumor cells and the staining intensity were evaluated to semi-quantitatively determine protein expression according to Li and Jiang [10] and Dian et al. [11]. The proportion of stained tumor cells was rated as no staining (−), <10 % staining (+), 11–50 % staining (++), 51–75 % staining (+++), and >75 % staining (++++). The staining intensity was classified as no staining, weak staining, moderate staining, and strong straining. Specimen with no straining, less than 10 % strained cells (+) or week straining were considered as negative expression, whereas other ones were as positive expression.

### Statistical analyses

All statistical analyses were performed using SPSS 20.0 (SPSS Inc, Chicago, IL, USA), and *P* < 0.05 was considered as statistically significant. Differences of the clinical characteristics between patients of groups A and B and expression of glycometabolism- and lipid metabolism-related proteins among the specimen of the three groups were analyzed by *χ*^2^ test. The clinicopathologic parameters were also analyzed in correlation to protein levels using *χ*^2^ test between groups A and B. Cox regression analysis (Enter method) was used to assess prognostic factors associated with progression-free survival (PFS) time and overall survival (OS) in groups A and B. The PFS rates were calculated using the Kaplan-Meier method and Kaplan-Meier survival curve was generated by log-rank test.

## Results

**Fig. 1:**
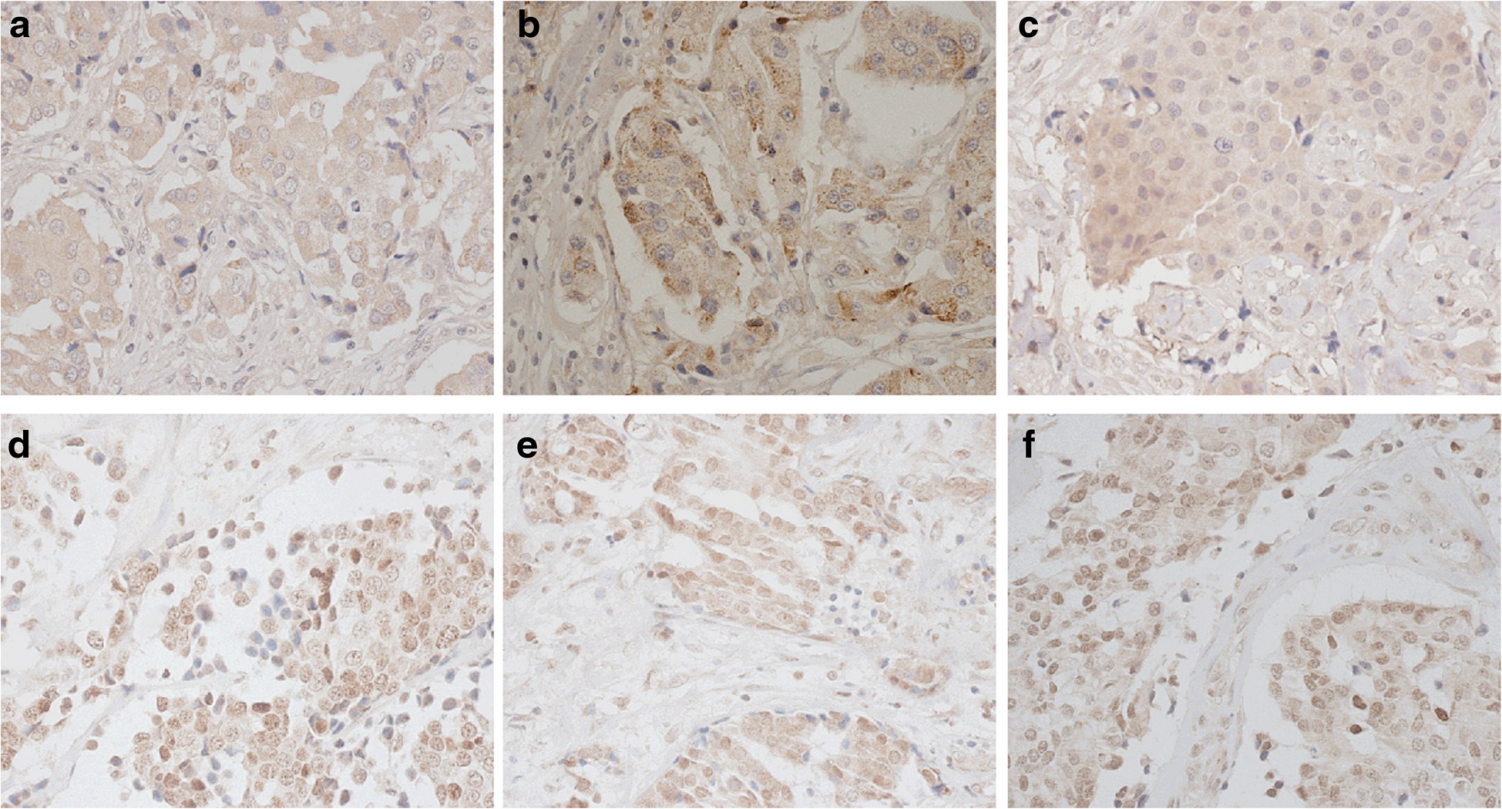
Microphotographs (×400) of immunohistochemical detection of lipid metabolism-related proteins consisting of CYP27A1 (**a**, **b**, and **c**) and CYP7B1 (**d**, **e** and **f**). **a**, **d** Primary breast carcinoma with recurrent metastasis during the follow-up. **b**, **e** Corresponding recurrent metastasis of breast carcinoma. **c**, **f** Primary breast carcinoma with no evidence of recurrence or metastasis

**Fig. 2:**
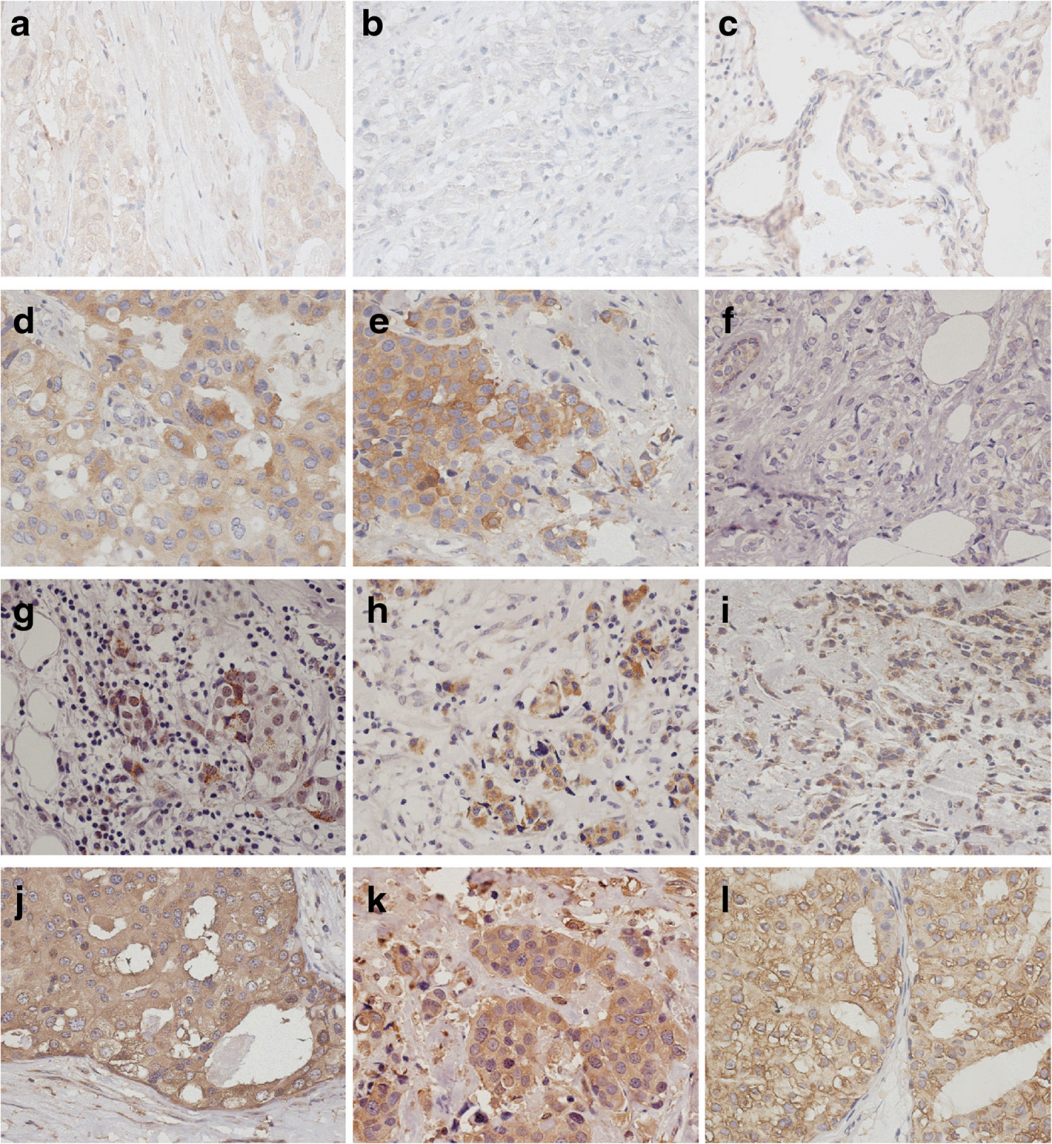
Microphotographs (×400) of immunohistochemical detection of glycometabolism-related proteins, including IGF-1 (**a**, **b**, and **c**), G6PD (**d**, **e**, and **f**), GSTP1 (**g**, **h**, and **i**), and PKM2 (**j**, **k**, and **l**). **a**, **d**, **g**, **j** Primary breast carcinoma with recurrent metastasis during the follow-up. **b**, **e**, **h**, **k** Corresponding recurrent metastasis of breast carcinoma. **c**, **f**, **i**, **l** Primary breast carcinoma with no evidence of recurrence or metastasis

The baseline characteristics of patients with primary breast carcinoma are shown in Table 1. Patients in groups A and B had similar age, lymph node metastasis, molecular subtypes, and pathological classification (all *P* > 0.05) except for the tumor staging and size (*P* = 0.043 and *P* < 0.001). Table 2 presents the expression of CYP27A1, CYP7B1, IGF-1, G6PD, GFPT1, and PKM2 proteins, which were all detected in the three groups of tumor tissues (Figs. 1 and 2). As shown in Table 2, G6PD was found to be highly expressed in the specimen of group A compared to that of groups B and A_M_, respectively (*P* = 0.006 and *P* = 0.025, respectively, data not shown). Interestingly, PKM2 expression was also significantly higher in the specimen of group A when compared to that of group A_M_ (*P* = 0.019, data not shown), but was similar with group B (*P* > 0.05). The specimens of the three groups had similar expression of CYP27A1, CYP7B1, IGF-1, and GFPT1 (all *P* > 0.05).

**Table 1:** Baseline characteristics of patients with breast carcinoma

Indicators	Group A (*n* = 10)	Group B (*n* = 10)	*P* value
Mean age (*n*)			0.972
≤60 years	7	7	
>60 years	3	3	
Lymph node metastasis (*n*)			0.881
0	2	2	
≤3	3	4	
>3	5	4	
Molecular subtypes (*n*)			0.639
Luminal A	3	4	
Luminal B	7	6	
HER-2	0	0	
Triple negative	0	0	
Tumor staging (*n*)			0.043
I	3	0	
II	2	7	
III	5	3	
Tumor size (cm)			<0.001
≤2 cm	10	2	
>2 cm	0	8	
Pathological classification (*n*)			0.136
IDC stage I	0	2	
IDC stage II	10	8	
Poorly differentiated adenocarcinoma	0	0	
Adenocarcinoma	0	0	
Metastatic adenocarcinoma	0	0	

Group A, primary breast carcinoma with recurrent metastasis during the follow-up; Group B, primary breast carcinoma with no pathological evidence of recurrence or metastasis; stage I, T1N0M0; stage II, T0-1N1M0, T2N0-1M0, and T3N0M0; stage III, T0-2N2M0, T3N1-2M0, T4N0-3M0, and T0-4N3M0; IDC, infiltrating duct carcinoma. *P* < 0.5 was considered statistically significant

**Table 2:** The expressions of CYP27A1, CYP7B1, IGF-1, G6PD, GFPT1, and PKM2 in tumor specimens of patients with breast carcinoma

	Group A (*n* = 10)	Group A_M_ (*n* = 10)	Group B (*n* = 10)	*P* value
CYP27A1 (*n*)				0.240
Negative	5	8	8	
Positive	5	2	2	
CYP7B1 (*n*)				0.585
Negative	0	1	1	
Positive	10	9	9	
IGF-1 (*n*)				0.315
Negative	8	8	10	
Positive	2	2	0	
G6PD (*n*)				0.010
Negative	3	8	9	
Positive	7	2	1	
GFPT1 (*n*)				0.082
Negative	8	10	6	
Positive	2	0	4	
PKM2 (*n*)				0.036
Negative	1	6	2	
Positive	9	4	8	

Group A, primary breast carcinoma with recurrent metastasis during the follow-up; Group A_M_, corresponding recurrent metastases; Group B, primary breast carcinoma with no pathological evidence of recurrence or metastasis. *P* < 0.5 was considered statistically significant

**Fig. 3:**
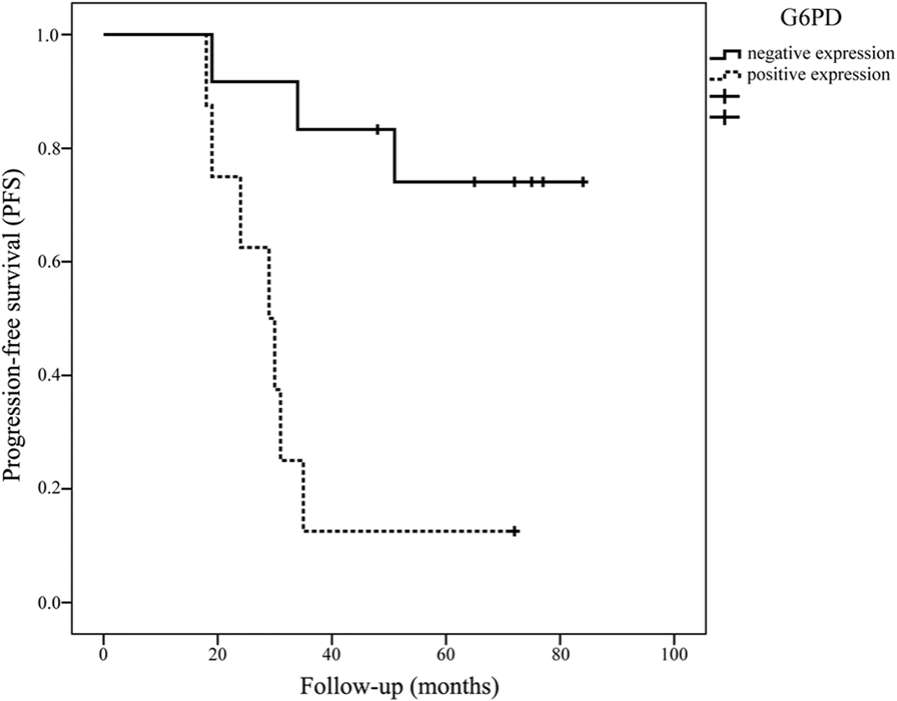
Kaplan-Meier survival curves of the patients with breast carcinoma according to the G6PD immunostaining results. Patients with G6PD-positive expression had shorter progression-free survival (PFS) time than those with G6PD-negative expression (log-rank test, *P* = 0.002)

Furthermore, Table 3 showed that the G6PD expression in tumor tissue was not associated with age, lymph node metastasis, molecular subtypes, tumor staging and size, as well as pathological classification (all *P* > 0.05). However, the PKM2 expression was relative to lymph node metastasis (*P* = 0.001). The variables with *P* < 0.05 between groups A and B were included in the Cox regression analysis (Enter method). Multivariate survival analysis indicated that only G6PD expression (hazard ratio = 13.488, *P* = 0.021) was an independent prognostic factor for mean PFS time (Table 4), suggesting that the primary breast carcinoma patients with upregulated G6PD expression were more likely to have poor PFS survival due to local recurrence or metastasis. However, PKM2 expressions in cancer tissue and tumor staging and size did not influence mean PFS time (all *P* > 0.05). Besides, all the variables had no effect on OS time (all *P* > 0.05). According to the Kaplan-Meier analysis, the mean PFS times of patients with G6PD-negative and G6PD-positive expression tumor were 71.36 ± 6.53 and 32.25 ± 5.67 months, respectively (Fig. 3, log-rank test, *P* = 0.002).

**Table 3:** Correlation between clinicopathological features and the protein expression of G6PD and PKM2 in patients with breast carcinoma

Indicators	G6PD		*P* value	PKM2		*P* value
	Negative	Positive		Negative	Positive	
Mean age (*n*)			0.111			0.891
≤0 years	10	4		2	12	
>60 years	2	4		1	5	
Lymph node metastasis (*n*)			0.763			0.001
0	2	2		3	1	
≤3	4	3		0	9	
>3	6	3		0	7	
Molecular subtypes (*n*)			0.444			0.948
Luminal A	5	2		1	6	
Luminal B	7	6		2	11	
Tumor staging (*n*)			0.058			0.277
I	0	3		1	2	
II	7	2		2	7	
III	5	3		0	8	
Tumor size (*n*)			0.070			0.306
≤2 cm	5	7		1	11	
>2 cm	7	1		2	6	
Pathological classification (*n*)			0.224			0.531
IDC stage I	2	0		0	2	
IDC stage II	10	8		3	15	

Stage I, T1N0M0; stage II, T0-1N1M0, T2N0-1M0, and T3N0M0; stage III, T0-2N2M0, T3N1-2M0, T4N0-3M0, and T0-4N3M0; IDC, infiltrating duct carcinoma. *P* < 0.5 was considered statistically significant

**Table 4:** Multivariate analyses of progression-free survival (PFS) and overall survival (OS) in patients with breast carcinoma

Variables	PFS			OS		
	*P* value	Hazard ratio (HR)	95 % CI	*P* value	HR	95 % CI
G6PD (negative vs. positive expression)	0.021	13.488	1.472–123.554	0.398	2.695	0.271–26.815
PKM2 (negative vs. positive expression)	0.835	1.304	0.107–15.868	0.674	1.916	0.093–39.686
Tumor staging						
I	0.095			0.977		
II	0.241	0.262	0.028–2.461	0.963	–	–
III	0.375	2.691	0.303–23.938	0.964	–	–
Tumor size (≤2 cm vs. >2 cm)	0.946	0.000	–	0.900	–	–

Cox regression analysis (Enter method) was used to assess prognostic factors associated with PFS and OS. A hazard ratio >1 with *P* < 0.05 indicates a greater likelihood of development of breast carcinoma, while a hazard ratio <1 with *P* < 0.05 indicates a lesser likelihood of development of breast carcinoma. A hazard ratio = 1 with *P* < 0.05 indicates that the given factor could not affect the development of breast carcinoma, but was significant in multivariate logistic regression model

## Discussion

Despite many reports regarding the prognostic factors of breast carcinoma [11–16], few studies have focused on the expression of various glycometabolism- and lipid metabolism-related proteins among primary breast carcinoma diagnosed with recurrent metastasis during the follow-up, the corresponding recurrent metastasis, and primary breast carcinoma with good prognosis yet. In the present study, we conducted a retrospective analysis on the expression of CYP27A1, CYP7B1, IGF-1, G6PD, GFPT1, and PKM2 proteins in cancer tissues from 20 cases of primary breast carcinoma, among whom 10 cases were diagnosed with recurrent metastasis and 10 cases had no evidence of recurrence or metastasis during their follow-up. The results showed that G6PD protein was significantly associated with prognosis in PFS.

G6PD, as the first rate-limiting enzyme of the pentose phosphate pathway (PPP), has been proved associated with the regulation of cell proliferation and transformation [17, 18]. The elevated G6PD activities were observed in various human cancers, such as renal cell carcinoma [19], bladder cancer [20], as well as gastric cancer [21]. In this study, we also identified an abnormally elevated expression of G6PD protein in primary breast carcinoma tissues with a positive follow-up of metastasis compared with corresponding recurrent metastases and primary breast carcinoma tissues with no evidence of recurrence or metastasis during follow-up, suggesting that G6PD overexpression might be responsible for cancer recurrent metastases. The elevated PKM2 expression in primary breast carcinoma diagnosed with recurrent metastasis relative to metastases might also reveal a possible inhibitive effect of low PKM2 expression on recurrent metastasis. However, this inhibitive effect was significantly affected by lymph node metastasis (*P* = 0.001), that is, the similar expression of PKM2 in primary breast carcinoma tissues with and without evidence of recurrent metastasis might be attributed to the similar lymph node metastasis between the two groups. In the work by Wang et al., G6PD protein in cancer tissue was found dependent on the tumor size and lymph node metastasis [21]; however, it was not witnessed in our study. The inconsistent results were probably due to the different cancer types and research conditions.

Furthermore, Cox multivariate analyses indicated that the G6PD protein was an independent prognostic factor for mean PFS, consistent with previous study. Patients with low expression of G6PD were more likely to live longer with no recurrence or metastasis, which was evidenced by the Kaplan-Meier survival curves (*P* = 0.002). Based on the above results, it seemed that a determination of G6PD expression before surgery might be of great importance in predicting the therapeutic effect and postoperative PFS for patients with primary breast carcinoma.

Several limitations to this study must be addressed. First, the cases of patients with recurrent metastasis were insufficient because the specimen were difficult to collect from recurrent metastases (most at infraclavicula and chest wall), and it might affect the statistical accuracy. Second, control comparisons within each patient (normal tissue vs. malignant tissue), HIF protein expression, and real-time reverse transcriptase polymerase chain reaction (RT-PCR) were not performed. However, under this perspective, our preliminary finding could lead to a broader line of research for further validation.

## Conclusions

In conclusion, this study did demonstrate that the overexpression of G6PD in primary breast carcinomas was associated with a high risk of recurrent metastasis and poor PFS. Further studies were certainly needed for this issue on a large number of patients with primary breast carcinomas and to clarify the role of G6PD protein or other else in breast cancer progression.
